# Inhibition of the NEDD8 conjugation pathway induces calcium-dependent compensatory activation of the pro-survival MEK/ERK pathway in acute lymphoblastic leukemia

**DOI:** 10.18632/oncotarget.23797

**Published:** 2017-12-31

**Authors:** Shuhua Zheng, Gilles M. Leclerc, Bin Li, Ronan T. Swords, Julio C. Barredo

**Affiliations:** ^1^ The Sheila and David Fuente Graduate Program in Cancer Biology, University of Miami Miller School of Medicine, Miami, FL, USA; ^2^ Department of Pediatrics, University of Miami Miller School of Medicine, Miami, FL, USA; ^3^ Department of Medicine, University of Miami Miller School of Medicine, Miami, FL, USA; ^4^ Department of Biochemistry and Molecular Biology, University of Miami Miller School of Medicine, Miami, FL, USA; ^5^ Sylvester Comprehensive Cancer Center, University of Miami Miller School of Medicine, Miami, FL, USA

**Keywords:** NEDDylation, pevonedistat, MEK/ERK signaling, store operated calcium entry, acute lymphoblastic leukemia (ALL)

## Abstract

*De novo* and acquired drug resistance and subsequent relapse remain major challenges in acute lymphoblastic leukemia (ALL). We previously identified that pevonedistat (TAK-924, MLN4924), a first-in-class inhibitor of NEDD8 activating enzyme (NAE), elicits ER stress and has potent *in vitro* and *in vivo* efficacy against ALL. However, in pevonedistat-treated ALL cell lines, we found consistent activation of the pro-survival MEK/ERK pathway, which has been associated with relapse and poor outcome in ALL. We uncovered that inhibition of the MEK/ERK pathway *in vitro* and *in vivo* sensitized ALL cells to pevonedistat. The observed synergistic apoptotic effect appears to be mediated by inhibition of the MEK/ERK pro-survival cascade leading to de-repression of the pro-apoptotic BIM protein. Mechanistically, Ca^2+^ influx via the Ca^2+^-release-activated Ca^2+^ (CRAC) channel induced protein kinase C β2 (PKC-β2) was responsible for activation of the MEK/ERK pathway in pevonedistat-treated ALL cells. Sequestration of Ca^2+^ using BAPTA-AM or blockage of store-operated Ca^2+^ entry (SOCE) using BTP-2 both attenuated the compensatory activation of MEK/ERK signaling in pevonedistat-treated ALL cells. Pevonedistat significantly altered the expression of Orai1 and stromal interaction molecule 1 (STIM1), resulting in significantly decreased STIM1 protein levels relative to Orai1. Further, we identified eIF2α as an important post-transcriptional regulator of STIM1, suggesting that pevonedistat-induced eIF2α de-phosphorylation selectively down-regulates translation of STIM1 mRNA. Consequently, our data suggest that pevonedistat potentially activates SOCE and promotes Ca^2+^ influx leading to activation of the MEK/ERK pathway by altering the stoichiometric Orai1:STIM1 ratio and inducing ER stress in ALL cells.

## INTRODUCTION

Acute lymphoblastic leukemia (ALL) is the most common cancer diagnosed in children and adolescents and has an overall 5-year event-free survival (EFS) of 80%, whereas adults with ALL have a 5-year EFS rate under 50% [1, 2]. For pediatric and adult patients who relapse, outcomes are particularly dismal [3]. In addition, long-term toxicity associated with current regimens leads to significant morbidity and mortality later in life. Therefore, there is a need to develop novel, more targeted strategies, that increase cure rates and mitigate against immediate and late toxicities.

In this context, we identified the NEDD8-activating enzyme (NAE) as a novel therapeutic target for ALL therapy, resulting in potent *in vitro* and *in vivo* anti-leukemic effects [4]¬. Protein NEDDylation is sequentially catalyzed by 3 enzymatic steps: activation of ubiquitin-like protein NEDD8 via its cognate E1 (NAE), transfer of NEDD8 to a NEDDylation specific E2 enzyme, and conjugation of NEDD8 to target proteins mediated by E3 ligase enzymes [5]. Most NEDD8 target proteins are cullins which are scaffold proteins for the Cullin-RING E3 Ligases (CRLs) [5]. NEDD8 conjugation is a prerequisite for full activation of CRLs which are key components of the ubiquitin-proteasome system (UPS) [5]. Pevonedistat was developed as a first-in-class inhibitor of NAE and inhibits CRL-mediated protein turnover [6]. In our previous study, we demonstrated pevonedistat induced apoptosis in ALL cells occurred by dysregulating cellular translation machinery causing induction of proteotoxic endoplasmic reticulum (ER) stress. Activation of both mTOR and UPR/eIF2α pathways [4]¬ were key mediators of this mechanism. Based on the increased vulnerability of ALL cells towards ER stress inducers [7, 8], our published data demonstrated that pevonedistat has potential for incorporation into ALL therapy [4]¬. We also showed that pevonedistat led to activation of the MEK/ERK pathway in ALL cells, and that co-targeting NEDDylation and MEK/ERK resulted in synergistic cell death [4]¬. We interpreted the induction of MEK/ERK as a compensatory survival mechanisms in response to pevonedistat cytotoxicity, but the mechanisms responsible for MEK/ERK activation remained unclear.

Overexpression of the MEK/ERK pathway has been associated with relapse and poor outcomes in pediatric ALL [9, 10], underscoring the clinical relevance of our findings above. In general, the MAPK cascade is activated by extracellular stimuli such as mitogens, growth factors, and cytokines that binds to receptor tyrosine kinases (RTK) at the cell surface to activate the small GTPase Ras protein. Activated MAPK signaling, represented by ERK1/2 phosphorylation, further regulates cell proliferation and survival [11, 12]. The rise of free intracellular calcium following binding of extracellular ligands to RTKs also influences the MEK/ERK signaling cascade [13]. In lymphocytes, increases in intracellular calcium are dependent on either Ca^2+^ ER stores or the store-operated Ca^2+^ entry (SOCE) mediated by the Ca^2+^-release-activated Ca^2+^ (CRAC) channel [14], with the latter identified as the main mechanism to replenish intracellular Ca^2+^ levels once the ER Ca^2+^ has been depleted [14]. In typical SOCE, depletion of Ca^2+^ stores activates the Ca^2+^ sensor proteins Stromal Interaction Molecules (STIM) 1 and 2, which translocate into the ER-plasma membrane (PM) junctions to activate the CRAC pore-forming subunit Orai1 [15, 16], in order to increase calcium influx. Given the importance of intracellular calcium in lymphocyte biology [13] and its role in inducing the MEK/ERK pathway activation [9, 10], we investigated the mechanistic role of SOCE/CRAC in pevo-induced activation of the MEK/ERK pathway in ALL cells.

## RESULTS

### Pevonedistat activates the PKC/MEK/ERK signaling cascade via Ca^2+^ mobilization

**Figure 1:**
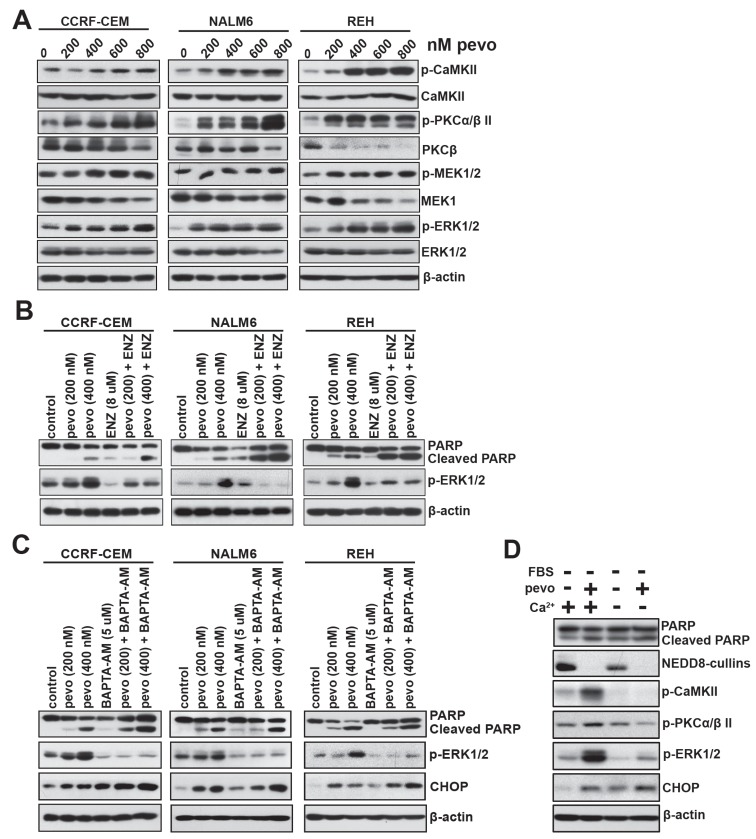
Activation of the MEK/ERK signaling cascade in pevonedistat-treated ALL cells is dependent on intracellular Ca^2+^ mobilization **A.** ALL cell lines CCRF-CEM (T-ALL), NALM6 (Bp-ALL) and REH (Bp-ALL) were treated with pevonedistat (200, 400, 600, and 800 nM) for 24 h. Protein extracts were probed for p-CaMKII (Thr286), CaMKII; p-PKCα/β II (Thr638/641), PKCβ, p-MEK1/2 (Ser217/221), MEK1/2, p-ERK1/2 (Thr202/Tyr204), and ERK1/2. **B.** Western blots of p-ERK1/2 (Thr202/Tyr204) expression in NALM6 cells treated with pevonedistat (200 and 400 nM) ± the PKC inhibitor enzastaurin (ENZ, 8 µM) for 24 h. **C.** Western blots of p-ERK1/2 (Thr202/Tyr204) and CHOP expression in CCRF-CEM, NALM6 and REH cells treated with pevonedistat (200 and 400 nM) ± the Ca^2+^ chelator BAPTA-AM (5 µM) for 24 h. **D.** Western blot analysis of p-CaMKII (Thr286), p-PKCα/β II (Thr638/641), p-ERK1/2 (Thr202/Tyr204), and CHOP expression in NALM6 cells cultured in Ca^2+^-free RPMI-1640 medium without FBS ± calcium (0.424 mM) for 24 h. PARP/Cleaved PARP expression was used as an indicator of apoptosis in the NALM6 cells treated above. β-actin levels served as loading controls and pevo stands for pevonedistat.

To probe and identify the mechanism(s) underlying MEK-ERK pathway activation in pevonedistat-treated ALL cells [4]. We first evaluated the role of the protein kinase C (PKCα/β II), a known mediator of MEK-ERK activation [17-19], in CCRF-CEM, NALM6, and REH cells treated with pevonedistat (200 to 800 nM). As shown in Figure 1A, pevonedistat significantly increased the cellular levels of p-PKCα/β II (Thr638/Thr641) and phosphorylated Ca^2+^/calmodulin-dependent protein kinase II (p-CaMKII) in all three ALL cell lines examined, which correlated with p-MEK1/2 (Ser217/Ser221) and p-ERK1/2 (Thr202/Tyr204) activation (Figure 1A). To test the causal relationship between increased p-PKCα/β II and MEK/ERK pathway activation, we treated these cell lines with the PKC inhibitor enzastaurin (ENZ; LY317615, [20]). Our data show that inhibition of PKC’s activity using ENZ significantly reduced the induction of p-ERK1/2 in pevonedistat-treated ALL cells (Figure 1B), establishing a cause-effect relationship between increased expression (activation) of p-PKCα/β II and MEK-ERK pathway activation in pevonedistat-treated ALL cells. Based on the established requirement for Ca^2+^ binding in the activation of PKCs [21] and the role of p-CaMKII in Ca^2+^ homeostasis [22], the observed increased cellular levels of p-CaMKII (Thr286) in all three ALL cell lines (Figure 1A) suggested that mobilization of Ca^2+^ may play a role in pevonedistat induced MEK/ERK activation. Consequently, we investigated the role of Ca^2+^ as a second messenger in PKC-induced MEK/ERK pathway activation in pevonedistat-treated ALL cells. For this purpose, we used the permeable-membrane calcium chelator BAPTA-AM [23] to examine its effects on the induction of p-ERK1/2. Consistent with our hypothesis, we found that the addition of BAPTA-AM significantly inhibited pevonedistat-induced p-ERK1/2 activation in all three ALL cell lines examined (Figure 1C), supporting a mechanistic role of Ca^2+^ in mediating MEK-ERK pathway activation in pevonedistat-treated ALL cells. As shown in Figure 1C, co-treatment with pevonedistat plus BAPTA-AM led to increased expression of CHOP compared to each agent alone, and correlated with greater induction of apoptosis in cells treated with both agents as demonstrated by increased PARP cleavage, suggesting that CHOP may also influence Ca^2+^ mobilization in pevonedistat-treated ALL cells.

To further assess and confirm the role of Ca^2+^ on MEK/ERK activation under conditions of pevonedistat-induced ER stress/UPR leading to CHOP activation, we cultured ALL cells in Ca^2+^ free RPMI-1640 medium and examined the expression of p-ERK1/2, p-PKCα/β II, and p-CaMKII following the addition of Ca^2+^ and/or pevonedistat. We found that the addition of Ca^2+^ to pevonedistat-treated NALM6 cells significantly up-regulated the expression of p-ERK1/2, p-PKCα/β II, and p-CaMKII, whereas its absence almost entirely abrogated their induction (Figure 1D). Based on these data, we concluded that the mechanism of PKC-mediated MEK/ERK pathway activation in pevonedistat-treated ALL cells is dependent on Ca^2+^ mobilization.

### Pevonedistat activates the Ca^2+^-release-activated Ca^2+^ (CRAC) channel

**Figure 2:**
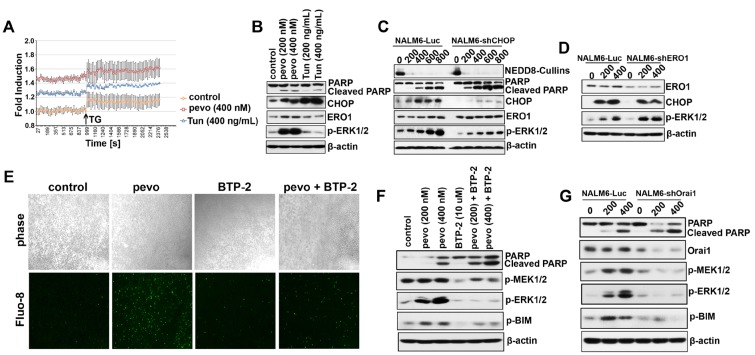
NEDDylation inhibition activated the CRAC channel to trigger Ca^2+^ influx **A.** Determination of intracellular Ca^2+^ levels in NALM6 cells treated with DMSO (control), pevonedistat (400 nM), or Tun (400 ng/mL) for 24 h using the the Ca^2+^ binding dye Fluo-8 AM. Absorption was measured at Excitation/Emission = 490/525 nm every 27 sec (mean ± SEM, n ≥ 3). 17 min post-treatment, thapsigargin (TG, 10 µM) was added to each well and cells incubated for 15 min at RT prior determination of intracellular Ca^2+^ levels. **B.** Western blot analysis of CHOP, ERO1, and p-ERK1/2 (Thr202/Try204) expression in NALM6 cells treated with pevonedistat (200 and 400 nM) and Tun (200 and 400 ng/mL) for 24 h. **C.** Western blot analysis of extracts from stably transfected NALM6 cells with lentiviral particles expressing the luciferase (NALM6-Luc) or shRNAs targeting CHOP (NALM6-shCHOP), and treated with pevonedistat (200, 400, 600, and 800 nM) for 24 h. **D.** Western blot analysis of the markers described in (B) expressed in stably transfected NALM6-Luc and NALM6-shERO1 cells and treated with pevonedistat (200 and 400 nM) for 24 h. **E.** Intracellular Ca^2+^ signal by Fluo-8 in NALM6 cells treated with pevonedistat (400 nM) ± the CRAC inhibitor BTP-2 (10 µM) for 24 h (10× magnification). **F.** Western blot analysis of MEK/ERK pathway proteins and p-BIM (Ser69) in NALM6 cells co-treated with pevonedistat (200 and 400 nM) ± BTP-2 (10 µM) for 24 h. **G.** Western blot analysis of p-MEK1/2 (Ser217/221), p-ERK1/2 (Thr202/Tyr204), p-BIM (Ser69), and Orai1 protein levels in NALM6-Luc and NALM6-shOrai1 stably transfected cells and treated with pevonedistat (200 and 400 nM) for 24 h. PARP/Cleaved PARP was used as apoptotic marker. β-actin as loading controls and pevo stands for pevonedistat.

To characterize the mechanism responsible for Ca^2+^ mobilization in pevonedistat-treated ALL cells, we performed a Ca^2+^ release assay using the Ca^2+^ binding dye Fluo-8 AM and determined the level of intracellular Ca^2+^ release in NALM6 cells treated with either vehicle (control), pevonedistat (400 nM), or the classical ER stress inducer tunicamycin (Tun, 400 ng/mL) known to stimulate Ca^2+^ release [24]. As previously described for this assay, following treatment with each agent, thapsigargin (TG, an inhibitor of the sarcoplasmic or endoplasmic reticulum Ca-ATPase family of calcium pump [25]) was added to augment ER Ca^2+^ release [26]. As shown in Figure 2A, both prior to and after the addition of TG, we found that pevonedistat induced greater Ca^2+^ release in NALM6 cells compared to cells treated with Tun or untreated controls. When we analyzed expression of p-ERK1/2 as a marker for activation of the MEK/ERK pathway, we observed induction of p-ERK1/2 only in pevonedistat-treated ALL cells correlating with the significantly greater increased in intracellular Ca^2+^ observed in pevonedistat-treated ALL cells (Figure 2B).

To further probe the mechanism of MEK/ERK activation in pevonedistat-treated ALL cells, we used lentiviral-based shRNAs to knockdown CHOP expression and block UPR-induced Ca^2+^ mobilization, and found that down-regulation of CHOP significantly attenuated the induction of p-ERK1/2 by pevonedistat (Figure 2C). Next, we examined the contribution of ER Oxidoreductin 1 (ERO1), a well-established downstream target of CHOP that mediates Ca^2+^ release from the ER [23], in the mechanism of Ca^2+^ mobilization and activation of p-ERK1/2 in pevonedistat-treated ALL cells. In ERO1 knock-down NALM6 cells (NALM6-shERO1), pevonedistat induced ERK1/2 phosphorylation was augmented compared to controls (NALM6-Luc) (Figure 2D), suggesting that UPR/CHOP/ERO1-induced Ca^2+^ release from the ER is likely only a minor contributor to p-ERK1/2 activation in pevonedistat-treated ALL cells. On this basis, we proceeded to investigate the role of the store-operated Ca^2+^ entry (SOCE), known to play a critical role in modulating Ca^2+^ influx in lymphocytes [14]. Since SOCE is principally mediated by the Ca^2+^-release-activated Ca^2+^ (CRAC) channel [12], we first examined its role in pevonedistat-induced Ca^2+^ release and p-ERK1/2 activation using the CRAC inhibitor BTP-2 (10 µM) in combination with pevonedistat (400 nM). By measuring the fluorescence intensity of Ca^2+^ binding Fluo-8 AM dye, we found that the addition of BTP-2 significantly inhibited the accumulation of intracellular Ca^2+^ in pevonedistat + BTP-2 treated NALM6 cells as compared to each agent alone or control (DMSO) (Figure 2E), and strongly reduced the activation of both p-MEK1/2 and p-ERK1/2, which correlated with increased cell death (cleaved PARP) (Figure 2F). In addition, we uncovered that inhibition of CRAC significantly down-regulated p-BIM (Ser69), a known pro-apoptotic marker that is inhibited through phosphorylation of its Ser69 residue by p-ERK1/2 to promote cell survival (Figure 2F) [27]. Taken together, these data support a role for CRAC rather than Ca^2+^ release from the ER, as the major mediator of pevonedistat-induced calcium influx in ALL cells. This role for CRAC was confirmed using shRNAs to down-regulate the expression of the CRAC pore-forming subunit Ora1, a central structural component of the CRAC Ca^2+^ channel entry, therefore preventing Ca^2+^ influx [12]. Indeed, knockdown Orai1 in NALM6 cells (NALM6-shOrai1) significantly abrogated the activation of p-MEK1/2 and p-ERK1/2 by pevonedistat, and concomitantly blocked the phosphorylated inhibition of BIM as compared to control cells (NALM6-Luc) (Figure 2G).

### The activation of CRAC is potentially mediated by pevonedistat-induced changes in the Orai1/STIM1 ratio

**Figure 3:**
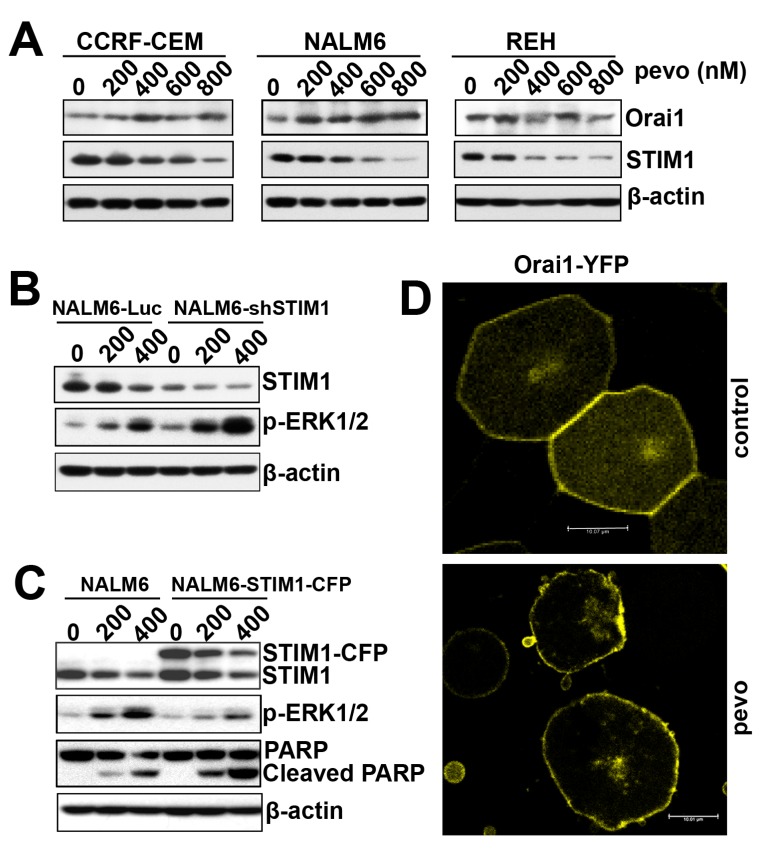
Pevonedistat alters the stoichiometric ratio between the CRAC’s pore-forming subunit Orai1 and the Ca^2+^ sensor protein STIM1 **A.** Western blot analysis of Orai1 and STIM1 proteins expressed in CCRF-CEM, NALM6, and REH ALL cells treated with pevonedistat (200, 400, 600, and 800 nM) for 24 h. **B.** Western blot analysis of STIM1 and p-ERK1/2 (Thr202/Tyr204) expression in stably transfected NALM6-Luc and NALM6-shSTIM1 expressing cells and treated with pevonedistat (200 and 400 nM) for 24 h. **C.** Western blot analysis of STIM1, p-ERK1/2 (Thr202/Tyr204), and PARP/Cleaved PARP (indicator of apoptosis) proteins expressed in nucleofected NALM6 cells with empty vector (NALM6) or STIM1-CFP (NALM6-STIM1-CFP), and treated with pevonedistat (200 and 400 nM) for 24 h. **D.** Confocal analysis for Orai1-YFP fluorescein expression in NALM6-Orai1-YFP cells treated with vehicle (DMSO, control) or pevonedistat (400 nM) for 24 h. β-actin was used as loading controls and pevo stands for pevonedistat.

Having established the role of CRAC and its pore-forming subunit Orai1 in pevonedistat-induced calcium influx and MEK/ERK activation in ALL, we examined the mechanistic interaction between Orai1 and the ER-membrane protein STIM1, which orchestrates Ca^2+^ influx by migrating to ER-plasma membrane appositions (puncta) to interact and open Orai1 [28]. It has recently been proposed that the activation of SOCE is regulated by the stoichiometric Orai1:STIM1 ratio, as opposed to their individual protein levels [29, 30]. Indeed, high STIM1 levels in cells traps Orai1, clusters it intracellularly, and prevents its plasma membrane enrichment. To test if the ratio of Orai1:STIM1 is altered by pevonedistat, we first determined their expression in pevonedistat-treated ALL cells and found that pevonedistat significantly down-regulated STIM1 expression in ALL cells whereas Orai1 expression was slightly upregulated (Figure 3A). The net effect of the observed protein expression changes was a significant alteration of the Orai1:STIM1 ratio in pevonedistat-treated ALL cells.

To investigate the regulatory role of the Orai1:STIM1 ratio on the activation of MEK/ERK signaling in pevonedistat-treated ALL cells, we examined the effects of down-regulating STIM1 on p-ERK1/2 activation by pevonedistat. Figure 3B shows that shRNA-mediated STIM1 knockdown in pevonedistat-treated NALM6 cells (NALM6-shSTIM1) resulted in greater expression of p-ERK1/2 compared to controls (NALM6-Luc), consistent with the hypothesis that pevonedistat-induced upregulation of p-ERK1/2 is mediated by changes in the Orai1:STIM1 ratio. The role of the Orai1/STIM1 ratio in this mechanism was further tested using NALM6 cells overexpressing STIM1 (NALM6-STIM1-CFP) which have an intrinsically altered Orai1:STIM1 ratio. Indeed, overexpression of STIM1-CFP in NALM6 cells abrogated the induction of p-ERK1/2 by pevonedistat and correlated with increased cleavage of PARP compared to wild-type controls, implicating changes in the Orai1:STIM1 ratio in pevonedistat-induced apoptosis of ALL cells (Figure 3C). The functional implication of this interaction was confirmed by confocal microscopy analysis of Orai1-YFP fluorescein expression in NALM6 cells treated with vehicle (control) or pevonedistat (400 nM). As shown in Figure 3D, the Orai1-YFP fluorescent signal was distributed evenly in the plasma membrane of control cells with undetectable intracellular localization, whereas that in pevonedistat-treated NALM6-Orai1-YFP cells, the fluorescent Orai1-YFP signal was mainly clustered in the plasma membrane with minimal intracellular localization, suggesting that pevonedistat-induced STIM1 downregulation may optimize the release of intracellularly trapped Orai1 to allow translocation into the plasma membrane. Taken together, these data suggest that pevonedistat altered the Orai1:STIM1 ratio potentially leading to CRAC activation with the expected increased Ca^2+^ influx, albeit other known mechanisms such as the induction of ER stress per se may also play a role in this cascade.

### Pevonedistat-induced de-phosphorylation of p-eIF2Α mediates STIM1 down-regulation

**Figure 4:**
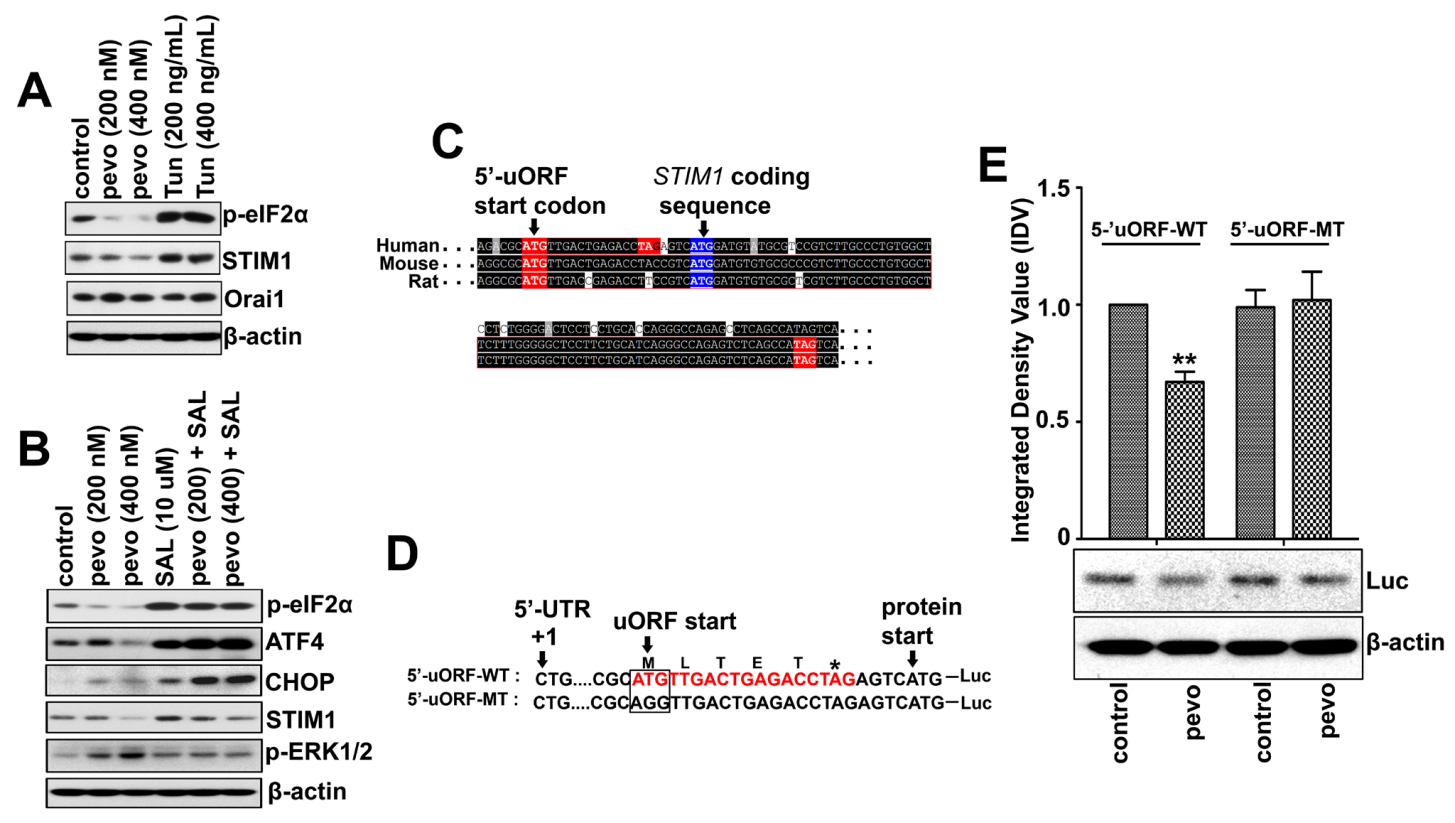
Pevonedistat down-regulates STIM1 expression *via* eIF2α-dependent translation of a short 5’-uORFsequence in ALL **A.** Western blot analysis of p-eIF2α (Ser51), STIM1, and Orai1 expression in NALM6 cells treated with pevonedistat (200 and 400 nM), or Tun (200 and 400 ng/mL) for 24 h. **B.** Western blot analysis of UPR regulatory proteins (ATF4, CHOP, and p-eIF2α (Ser51)), STIM1, and p-ERK1/2 (Thr202/Tyr204) expression in NALM6 cells treated with pevonedistat (200 and 400 nM) and SAL (10 µM) either alone or in combination for 24 h. **C.** Alignment of 5’-UTR DNA sequences from human, mouse, and rat STIM1 genes obtained from the RefSeq database (https://www.ncbi.nlm.nih.gov/refseq). The initiation start (ATG) and stop (TAG) codons within the 5’-uORF sequences are indicated in red color. The start codon for STIM1 mRNA is marked in blue. **D.** Nucleotide sequences of DNA duplexes containing either the wild-type (5’-uORF-WT) or the mutated (5’-uORF-MT) 5’-uORF STIM1 region that were cloned in-frame to the luciferase coding gene (Luc) in the pGL3-Enhancer plasmids. The initiation codon ATG of the putative 5’-uORF was mutated to AGG (5’-uORF-MT). The STIM1 mRNA +1 and initiating methionine codon are indicated. **E.** Western blot analysis of the luciferase expressed in NALM6 cells nucleofected with either plasmids pGL3-5’-uORF-WT or pGL3-5’-uORF-MT, and treated with pevonedistat (400 nM) for 24 h. The integrated density value (IDV) of each immuno-detected luciferase bands were normalized to their respective β-actin level and expressed relative to control (untreated NALM6/5’-uORF-WT). Pevo stands for pevonedistat.

The fact that pevonedistat induced UPR-mediated cell death via de-phosphorylation of p-eIF2α (Ser51) in ALL cells, altering the UPR regulation of protein translation [4], prompted us to examine its role in pevonedistat-induced CRAC/MEK/ERK pathway activation. As previously shown, p-eIF2α regulates the translation of certain ER stress response genes such as CHOP and ATF4 [31, 32], and under ER stress conditions eIF2α is phosphorylated to inhibit global protein translation except for a specific class of mRNAs containing a short upstream open reading frame (5’-uORF) selected for enhanced translation. To test if STIM1 mRNA may be regulated by a similar mechanism, we compared the level of STIM1 expression in NALM6 cells treated with pevonedistat and the ER stress inducer Tun. We found that STIM1 expression correlated with p-eIF2α levels: both were down-regulated by pevonedistat and conversely up-regulated in Tun-treated cells (Figure 4A). We then examined the effects of blocking eIF2α de-phosphorylation on STIM1and p-ERK1/2 expression in NALM6 cells treated with the eIF2α inhibitor salubrinal (SAL, 10µM) [33] ± pevonedistat (200 and 400 nM). As discussed above, treatment with pevonedistat down-regulated p-eIF2α, even in presence of SAL. We found that the addition of SAL either alone or in combination with pevonedistat resulted in higher levels of STIM1 expression compared to untreated cells (Figure 4B). CHOP and ATF4 expression, known to be regulated by p-eIF2α were used as positive controls. More important, SAL up-regulated STIM1 and the addition of pevonedistat to SAL-treated cells resulted in lower STIM1 and p-ERK1/2 levels compared to cells treated with pevonedistat alone, supporting a role for eIF2α in STIM1 down-regulation leading to CRAC-induced calcium influx (Figure 4B).

Next, we screened the RefSeq database (www.ncbi.nlm.nih.gov/refseq) for putative 5’-uORF regulatory sequences within human, mouse and rat STIM1 genes, and identified only one 5’-uORF conserved sequence among these three species (Figure 4C). The function of this putative 5’-uORF in the eIF2α-dependent translation of STIM1 mRNA was determined using duplexed oligonucleotide fragments containing either wild-type (ATG, 5’-uORF-WT form) or mutated 5’-uORF initiation codon (ATG substituted to AGG, 5’-uORF-MT form) that were cloned into the pGL3-Ehancer plasmid to generate an in-frame fusion with the luciferase coding gene (plasmids pGL3-uORF-WT and pGL3-uORF-WT). (Figure 4D). As shown in Figure 4E, NALM6 cells expressing the putative wild-type 5’-uORF-WT form exhibited lower level of luciferase expression when treated with pevonedistat as compared to controls, whereas no difference was observed between control and pevonedistat-treated NALM6 cells expressing the mutated 5’-uORF-MT form. Therefore this putative STIM1 5’-uORF may play a partial role in the translational regulation of STIM1 mRNA, as it has been shown that more than one 5’-uORF may be required for a more robust regulation seen with other eIF2α-dependent genes [34].

### Activation of the MEK/ERK pathway by pevonedistat protects ALL cells from apoptotic cell death by phosphorylating/inhibiting BIM activity

**Figure 5:**
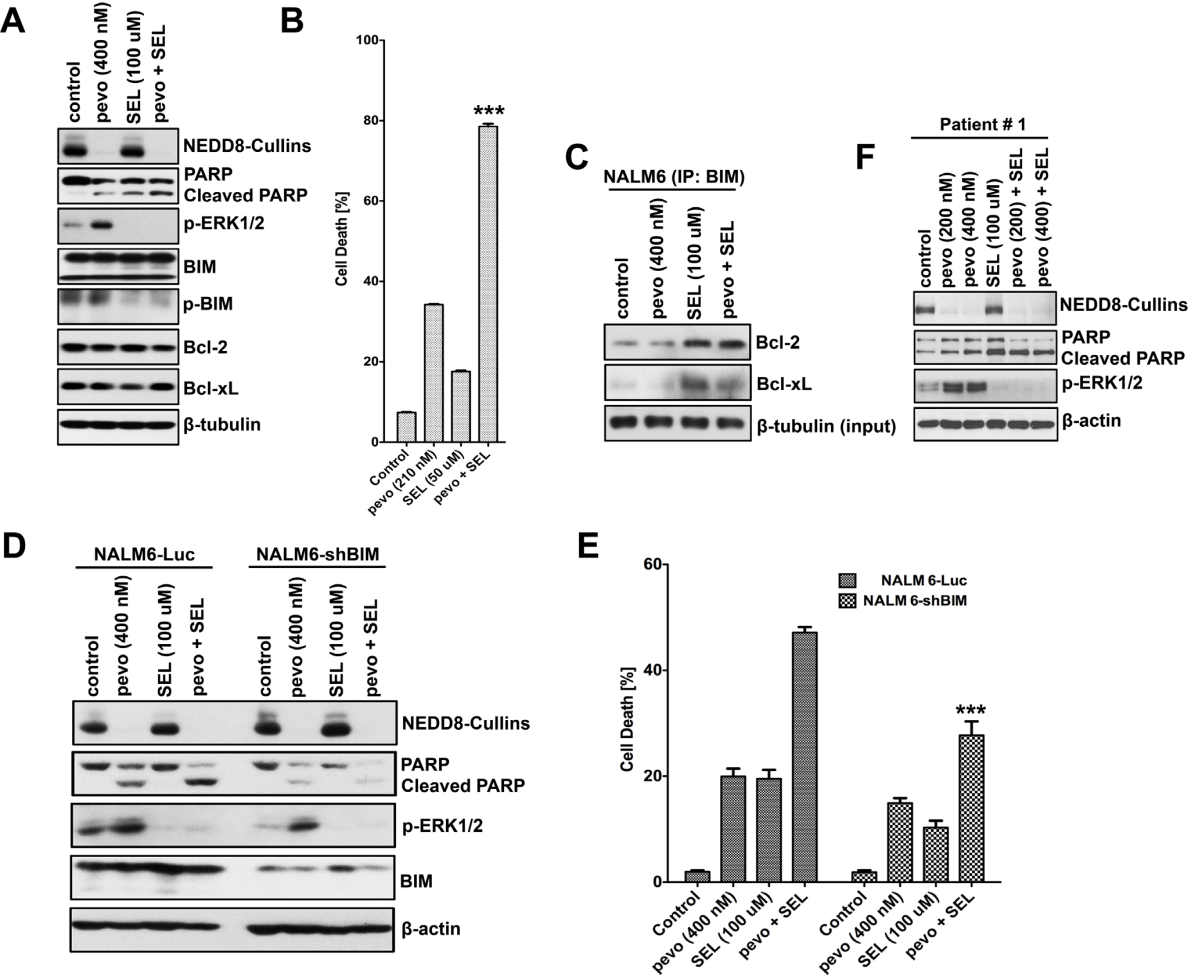
Selumetinib synergistically sensitize ALL cells to pevonedistat *via* inhibition of the MEK/ERK/p-BIM pro-survival pathway **A.** Western blot analysis of p-ERK (Thr202/Tyr204), BIM, p-BIM (Ser69), and anti-apoptotic proteins Bcl-2 and Bcl-xL expressed in NALM6 cells co-treated with pevonedistat (400 nM) and SEL (100 µM) for 24 h. **B.** Determination of cell death in NALM6 cells co-treated with pevonedistat (210 nM) and SEL (50 µM) for 72 h using trypan blue exclusion assays. Cell death is expressed as a percentage of the number of dead cells in the population (mean ± SEM, *n* = 3). *p* < 0.0001 for the combination pevonedistat + SEL *vs.* each single agent. **C.** Co-IPs analysis of the binding interactions between BIM with the anti-apoptotic proteins Bcl-2 and Bcl-xL in NALM6 cells treated with pevonedistat (400 nM) and SEL (100 µM) either alone or in combination for 24 h. Cellular extracts were immuno-precipitated with BIM antibodies, subjected to SDS-PAGE, and probed with Bcl-2 and Bcl-xL antibodies. **D.** Western blot analysis of NEDD8-Cullins, p-ERK1/2 (Thr202/Tyr204), and BIM proteins in stably transfected NALM6-Luc and NALM6-shBIM expressing cells, and treated with pevonedistat (400 nM) and SEL (100 µM) either alone or in combination for 24 h. **E.** Determination of cell death in NALM6-Luc and NALM6-shBIM cells described in (D). Cell death is expressed as a percentage of the number of dead cells in the population (mean ± SEM, *n* = 3). ***, *p* < 0.001 for pevonedistat plus SEL *vs.* controls. **F.** Western blot analysis of NEDD8-Cullins, and p-ERK1/2 (Thr202/Tyr204) expression in primary ALL cells, and treated with pevonedistat (200 and 400 nM) ± SEL (200 and 400 µM) for 24 h. PARP/Cleaved PARP was used as marker for apoptosis. Levels of β-actin and β-tubulin were used as loading controls. Pevo stands for pevonedistat.

We previously demonstrated that co-targeting the NEDD8-conjugation and MEK/ERK pathways led to synergistic *in vitro* and *in vivo* cell death in ALL cells [4], but the mechanism remained unclear. To begin to understand the mechanism underlying this synergistic effect, we first evaluated the cytotoxicity and the expression of pro- and anti-apoptotic Bcl-2 family proteins in NALM6 cells co-treated with pevonedistat and the MEK inhibitor selumetinib (SEL). We found lower expression of p-BIM (Ser69) in cells treated with SEL either alone or in combination with pevonedistat (Figure 5A), whereas no difference in expression was observed for total-BIM, Bcl-2 and Bcl-xL proteins. More important, the level of p-BIM (Ser69) was almost completely abrogated by the combination pevonedistat + SEL compared to pevonedistat alone, and p-BIM and pERK1/2 down-regulation correlated with higher PARP cleavage and cell death (Figures 5A, 5B). Our data is consistent with the well-established role of the MEK/ERK pathway in promoting cell survival via phosphorylation/inhibition of BIM at Ser69 by altering its ability to bind other anti-apoptotic Bcl-2 proteins [27, 35]. To evaluate the role of BIM in this synergistic mechanism, we used Co-IP to determine the interaction of BIM with Bcl-2 and Bcl-xL in pevonedistat ± SEL treated ALL cells. Figure 5C demonstrates that the binding between BIM and the anti-apoptotic proteins Bcl-2, and Bcl-xL was significantly enhanced in cells treated with SEL alone or in combination with pevonedistat as compared to controls or pevonedistat alone treated cells. These data support a major role for BIM in promoting cell death by neutralizing Bcl-2 and Bcl-xL anti-apoptotic activity. BIM’s role was confirmed with shRNA-mediated BIM knock-down (NALM6-shBIM) (Figure 5D). We found significantly less cell death in BIM knock-down cells, evidenced by PARP cleavage (Figure 5D) and cell death assays (p < 0.001 for pevonedistat plus SEL vs. controls) (Figure 5E), treated with the combination pevonedistat + SEL compared to controls (NALM6-Luc). Similar effects were observed in primary ALL cells co-treated with pevonedistat + SEL (Figure 5F), highlighting the clinical relevance of our findings. Taken together, our data show that inhibition of the MEK/ERK pathway by SEL, which prevents phosphorylation and inhibition of the pro-apoptotic protein BIM, is critical to the synergistic effect observed in pevonedistat + SEL treated ALL cells.

### Pevonedistat plus selumetinib induces significant *in vivo* anti-leukemic activity in the NSG ALL mouse model

**Figure 6:**
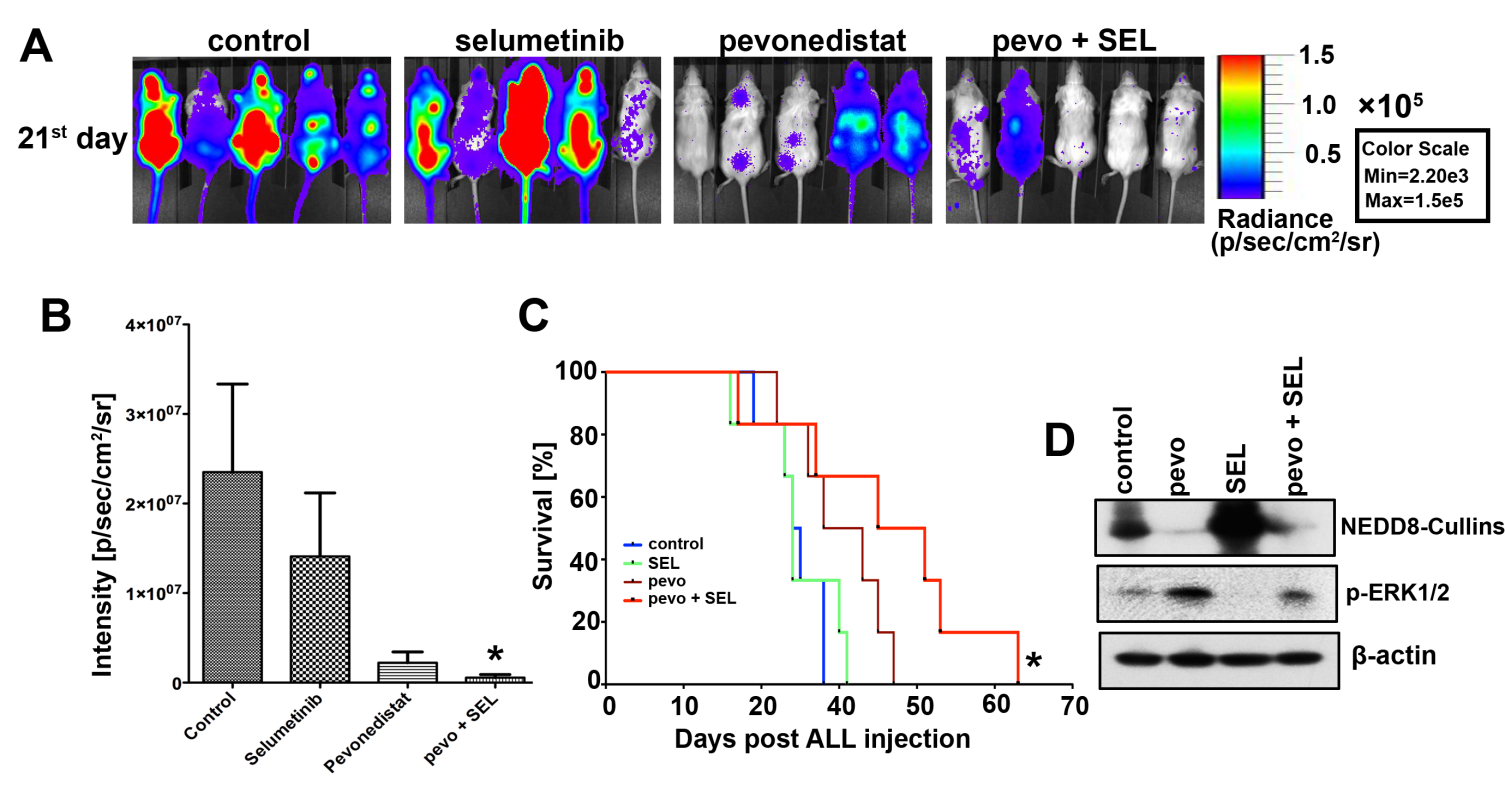
Pevonedistat plus selumetinib reduced tumor burden and prolonged the survival of NSG mice engrafted with ALL **A.** NSG mice were injected via the tail vein with 10^6^ viable human NALM6-Luc cells. Once engraftment confirmed, animals were assigned to four treatment groups of 6 mice each, treated for 10 days (21^st^ day post ALL injection) with either vehicles (control), pevonedistat (s.c., 66 mg/kg); SEL (p.o., 50 mg/kg) or the combination pevonedistat plus SEL, and imaged in the IVIS facility. **B.** Signal intensity from the imaging were quantified, compared, and adjusted to p/sec/cm^2^/sr. *, *p* < 0.05, for signal intensity in pevonedistat plus SEL *vs.* vehicles-treated mice (control). **C.** Kaplan-Meier curves for ALL engrafted NSG mice treated with the compounds described above. *, *p* < 0.05, for the survival rate of pevonedistat plus SEL treated mice *vs.* vehicles-treated mice (control). **D.** Western blot analysis of NEDD8-Cullins, and p-ERK1/2 (Thr202/Tyr204) expression in NALM6-Luc cells extracted from the spleens of deceased mice from each treatment groups. β-actin was used as loading controls. Experiments were conducted under the supervision of the University of Miami’s Institutional Animal Care and Use Committee. Pevo stands for pevonedistat.

The *in vivo* relevance of the combination pevonedistat plus SEL was evaluated using our NSG ALL mouse model in which NSG mice are engrafted with NALM6 luciferase expressing cells (NALM6-Luc) [4]. As previously described [4], following confirmation of ALL cells’ engraftment in NSG mice, animals were assigned to four treatment groups of 6 mice each: treatment with pevonedistat (s.c., 66 mg/kg) or SEL (p.o., 50 mg/kg) alone or in combination, as well as vehicle-treated controls. Treatment was administered twice daily on weekdays and once per day on weekends and bioluminescence analysis of animals was conducted starting at day 21 days post-engraftment. This analysis demonstrated a trend to decrease tumor burden in mice treated with pevonedistat alone and a statistically significant reduction of tumor burden in mice treated with the combination (p < 0.05 for pevonedistat + SEL vs. control) (Figure 6A and 6B). Kaplan-Meier analysis also showed a trend of improved survival in mice treated with pevonedistat alone and in combination over the control group (p < 0.05) (Figure 6C). In order to assess if this survival advantage is an on-target effect, NALM6-Luc cells were harvest from the spleen of engrafted-NSG mice and analyzed for changes in NEDD8-Cullins and p-ERK1/2 expression. We found consistent *in vivo* inhibitory effects of pevonedistat on the NEDDylation of cullins, and activation of p-ERK1/2 in the NALM6-Luc cells harvested from mice treated with pevonedistat ± SEL, and these findings were comparable to the *in vitro* data obtained with ALL cell lines and primary ALL cells [4]. Therefore, the activity of the combination of pevonedistat plus SEL *in vivo* mimics the more extensive analysis conducted *in vitro* using ALL cell line models and patient-derived samples, highlighting the potential clinical relevance of our findings.

## DISCUSSION

This study identified the Ca^2+^ release-activated Ca^2+^ (CRAC) channel [14] as the major mechanism responsible for activation of the pro-survival MEK/ERK signaling cascade following NEDD8-conjugation pathway inhibition in ALL cells. We uncovered that pevonedistat mobilized intracellular Ca^2+^ via activation of the store-operated Ca^2+^ entry (SOCE) leading to augmentation of the CRAC-mediated Ca^2+^ influx, and activation of the PKC/MEK/ERK signaling cascade. This is evidenced by direct measurement of intracellular calcium using the Ca^2+^ binding Fluo-8 AM dye, and the CRAC inhibitor BTP-2 which clearly demonstrated that pevonedistat increased Ca^2+^ influx in ALL cells, and that activation of p-ERK1/2 was significantly reduced in presence of the Ca^2+^ chelator BAPTA-AM or the PKC inhibitor enzastaurin. Furthermore, Ca^2+^ influx was shown to be triggered by changes in the stoichiometric ratio between the CRAC’s pore-forming subunit Orai1 and the Ca^2+^ sensor protein STIM1 [15, 16] [29, 30]. Our data support this model and indicate that pevonedistat treatment altered the Orai1:STIM1 ratio, which together with ER stress, contributes to CRAC-dependent PKC/MEK/ERK activation. More importantly, we demonstrate for the first time that STIM1 mRNA expression appears at least partially regulated by a post-transcriptional mechanism involving eIF2α-dependent translation of a short upstream open reading frame sequence (5’-uORF). Normally under ER stress, eIF2α is phosphorylated by the (ER)-resident protein kinase PERK to block global protein synthesis except for a select group of mRNAs that requires eIF2α phosphorylation for enhanced expression such as the stress response genes CHOP and ATF4 [31, 32, 36, 37]. These stress response genes have been found to be regulated by translation of a series of short 5’unstranslated regions [37]. On this basis, the STIM1 mRNA may be regulated by a similar mechanism. Our data clearly demonstrated that inhibition of p-eIF2α de-phosphorylation using the eIF2α inhibitor salubrinal resulted in higher levels of STIM1, CHOP and ATF4 expression, and significantly abrogated the induction of p-ERK1/2 in pevonedistat-treated ALL cells. In addition, we showed that mutation of the ATG initiation codon of the STIM1 5’-uORF to AGG significantly reduced the translation of this 5’-ORF as evidenced by lessened expression of the in-frame luciferase reporter gene, indicating that the putative 5’-uORF STIM1 is functional and may play a role in regulating the translation of STIM1 mRNA. Through this translational regulatory mechanism, STIM1 mRNA level changes in response to stress conditions to evade global repression of translation mediated by eIF2α as part of an integrated stress response signaling critical for cellular adaption and survival. This mechanism is essential in development, immunity, auto-immunity, neuro-degeneration, and cancer [37], and through STIM1 expression, it adds to the complexity of Ca^2+^ homeostasis regulation as a second messenger in ALL cells’ response to stress conditions.

**Figure 7:**
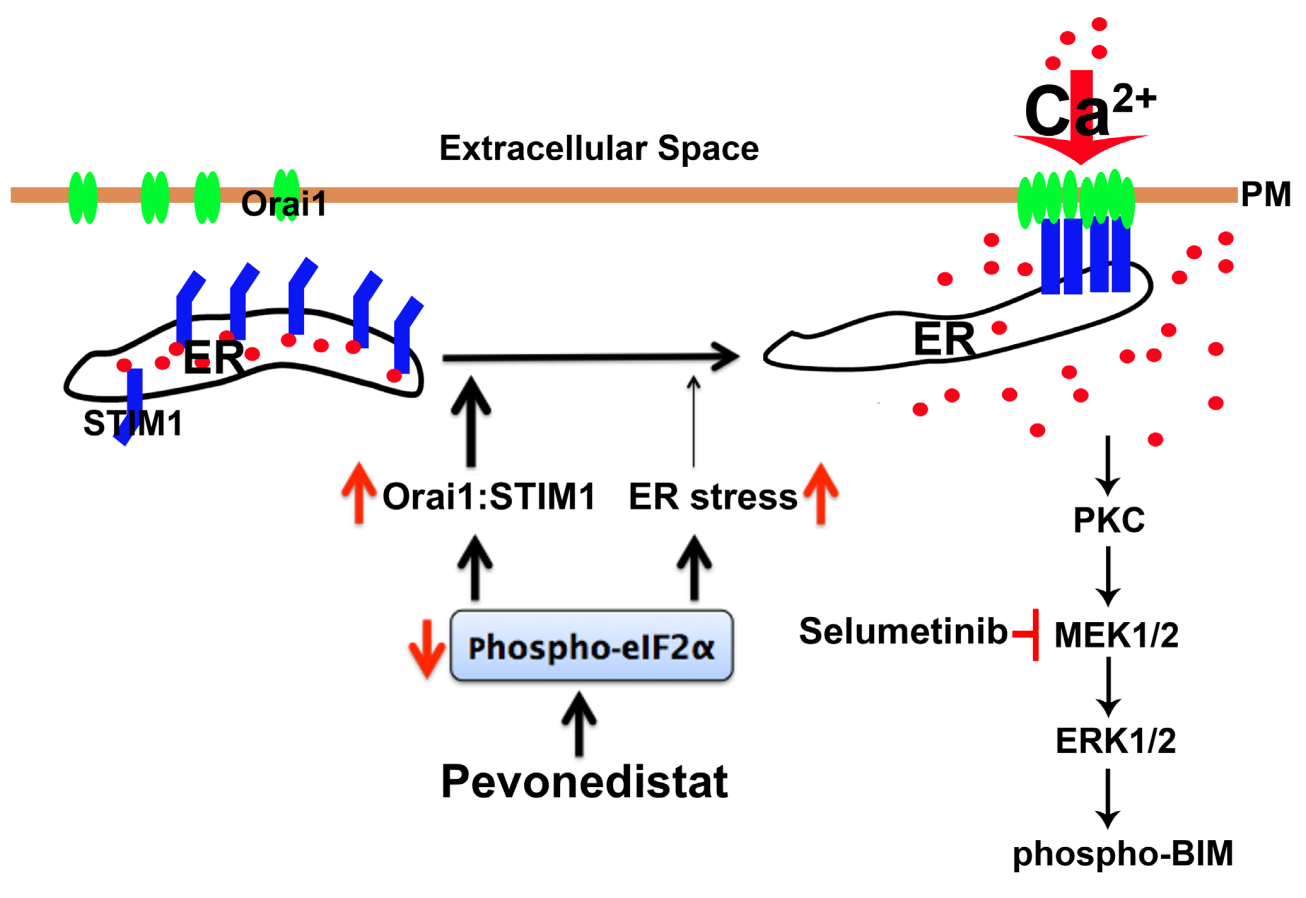
Proposed model of the role of SOCE/CRAC in pevonedistat-induced activation of the PKC/MEK/ERK survival pathway in ALL cells NEDDylation inhibition by pevonedistat induces eIF2α de-phosphorylation which reduces the cellular level of STIM1 and in turn changes the stoichiometric ratio between Orai1:STIM1, leading to CRAC activation and concomitant increase of Ca^2+^ influx. De-phosphorylation of eIF2α also contributes ER stress which may also contribute to activation of SOCE with pevonedistat-mediated NEDDylation inhibition in ALL cells. Pevonedistat-induced mobilization of intracellular Ca^2+^ will activated PKC and its downstream pro-survival MEK/ERK signaling cascade to promote phosphorylation and inhibition of the pro-apoptotic BIM activity. Treatment with SEL will inhibit the MEK/ERK pathway, and promote de-phosphorylation of BIM to increase its pro-apoptotic activity, resulting in synergistic cell death in pevonedistat-treated ALL cells.

It has been shown that in cells with high STIM1 levels, Orai1 is trapped with STIM1 intracellularly, preventing its plasma membrane enrichment. On the other hand, when STIM1 levels are low or down-regulated, Orai1 is released from the STIM1 intracellular clusters and localized to the plasma membrane to trigger Ca^2+^ influx [29]. In support of this mechanistic role for STIM1 in Ca^2+^ regulation in pevonedistat-treated ALL cells, we showed that in NALM6 cells overexpressing Orai1-YFP, the fluorescent signal was distributed evenly in the plasma membrane with undetectable intracellular localization, whereas in the pevonedistat-treated NALM6-Orai1-YFP expressing cells the signal was clustered in the plasma membrane with minimal intracellular localization. These findings are consistent with pevonedistat-induced alteration of the Orai1:STIM1 ratio leading to CRAC activation and increased Ca^2+^ influx, a critical event required for activation of the PKC/MEK/ERK pathway. Consequently, we propose that de-phosphorylation of p-eIF2α by pevonedistat will not only lead to ER stress/UPR-mediated cell death via increased protein translation in ALL as previously reported [4], but also enhances the translation rate of the short STIM1 5’-uORF resulting in down-regulation of STIM1 gene expression and alteration of the Orai1:STIM1 ratio. This stoichiometric changes will activate the CRAC channel, and increase Ca^2+^ influx to subsequently activate the compensatory survival PKC/MEK/ERK pathway as depicted in the proposed model (Figure 7). However, as also indicated STIM1 down-regulation contributes to SOCE activation and MEK/ERK activation but other yet to be identified mechanism(s) likely participate in the induction of cell death and MAPK activation. In the ER, Ca^2+^ is required for proper protein folding [38], and it is replenished by activation of the CRAC channel. Thus, it is conceivable that during typical ER stress (e.g., cells treated with the classical ER stressor tunicamycin [24]), the UPR/PERK will shut down global protein synthesis by promoting eIF2α phosphorylation, which selectively up-regulates translation of mRNAs that harbor small 5’-uORF such as STIM1 [34]. However, additional evidence is required to definitively substantiate the role of eIF2α in the regulation of CRAC-mediated calcium influx in ALL.

The cellular relevance of these molecular signaling interactions leading to increased Ca^2+^ influx in pevonedistat-treated ALL cells is manifested by the downstream activation of the pro-survival MEK/ERK pathway. Recently, Zhou et al. [39] reported that pevonedistat induced p-ERK1/2 expression in solid tumors via activation of the epidermal growth factor receptor (EGFR). We tested this mechanism and found no role for EGFR in pevonedistat-induced p-ERK1/2 activation in ALL cells, even though EGFR was expressed in NALM6 cells (data not shown). Herein, we demonstrated that activation of PKC/MEK/ERK signaling cascade was dependent on the induction of CRAC-mediated Ca^2+^ influx, and that co-targeting the NEDD8 conjugation pathway plus the MEK/ERK pathway led to *in vitro* [4] and *in vivo* synergistic cell death (Figure 7). Aberrant activation of the MEK/ERK pathway has been associated with drug resistance, relapse and poor outcome in pediatric ALL [9, 10, 35]. Here, we clearly show that the synergistic efficacy observed in ALL cells co-treated with pevonedistat plus SEL is due to inhibition of the MEK/ERK pathway leading to increased activity of the pro-apoptotic BIM protein. This is evidenced by our data showing decreased p-BIM expression and greater cell death in pevonedistat + SEL treated ALL cells, together with Co-IP experiments demonstrating that changes in the interaction between BIM and Bcl-2 or Bcl-xL correlate with cell death. It is well established that the MEK/ERK pathway regulates the activity of many proteins involved in apoptosis including Bcl-2, Mcl-1, Bad, caspase-9, and Bim [35]. Mechanistically, the activation of the ERK1/2 pro-survival signaling pathway was shown to promote phosphorylation and proteasome-dependent degradation of the BH3-only protein Bim [40]. Considering that BIM is a known target of CRLs [41], it is possible that simultaneous inhibition of the NEDD8 conjugation pathway and the MEK/ERK signaling leading to stabilization of BIM and increased BIM pro-apoptotic activity, respectively, will result in synergistic ALL cell death. Our findings support the role of BIM as a key mediator of cell death in pevonedistat-treated ALL [42], and CCL cells [43].

Our pre-clinical data show significant synergistic effect of pevonedistat and SEL in ALL, and suggest a clear path for future translation into the clinic for both children and adults with ALL. Indeed, we are initiating a clinical trial of pevonedistat in combination with reinduction chemotherapy for adolescents and young adults with refractory/relapsed ALL (IND 136393; ClinicalTrials.gov 20170602). Successful treatment strategies for ALL continue to require the use of multi-agent regimens, and given that multi-agent therapy is still important in patients with relapse, a favored strategy for the introduction of new agents into ALL therapy has been to incorporate promising new agents into established multi-agent platforms, such as vincristine, prednisone (frequently replaced by dexamethasone in practice), L-asparaginase, and doxorubicin (VPLD). An example of this strategy is the successful incorporation of bortezomib into VPLD therapy for ALL recently reported by Messinger et al. [44]. We previously demonstrated *in vitro* and *in vivo* synergy between pevonedistat and dexamethasone in ALL [4]. The first clinical report of pevonedistat in patients with myeloid neoplasms was published recently [45]. In this clinical trial, the safety profile and minimal myelosuppressive effects of this agent increase enthusiasm for pevonedistat combination studies in ALL. Here, we showed that co-treatment of NSG mice harboring human ALL (NALM6-Luc) with pevonedistat plus the MEK/ERK inhibitor selumetinib had lower disease burden and statistically significant increased survival when compared to either agent alone, lending support to further investigate the mechanism of synergy between agents targeting these two pathways. Given that aberrant MEK/ERK activation is associated with resistance and relapses in pediatric ALL [35], our study provide a rationale to further evaluate the incorporation of drugs targeting NEDDylation either alone or in combination with agents that target the MEK/ERK signaling pathway as part of a multi-agent approach for ALL therapy. Indeed, a clinical trial for relapsed/refractory ALL combining standard VXLD induction with pevonedistat is currently under development, and a subsequent trial could explore the combination of pevonedistat plus agents that inhibit the MEK/ERK pathway.

## MATERIALS AND METHODS

### Cell culture and reagents

RPMI-1640 medium (Cellgro), supplemented with 10% heat-inactivated fetal bovine serum (FBS) (Cellgro) and antibiotics (penicillin, 100 IU/mL; streptomycin, 100 μg/mL; Cellgro), was used to culture the ALL cell lines CCRF-CEM (T-ALL), NALM6 (Bp-ALL), REH (Bp-ALL) at 37 °C with 5% CO2. Stably transfected NALM6 cells expressing the luciferase gene (NALM6-Luc) were generated as described [4]. Primary ALL cells were co-cultured with human bone marrow stromal cell feeder as described [8]. Pevonedistat was provided by Millennium Pharmaceuticals, Inc (Cambridge, MA). Selumetinib, and enzastaurin (LY317615) were purchased from Selleckchem (Houston, TX). BTP-2 and BAPTA-AM were obtained from Abcam (Cambridge, MA), and luciferin from Perkin Elmer (Waltham, MA).

### Cell viability and cell death assays

Cell viability and cell death were measured with trypan blue exclusion using the Vi-CELL XR analyzer (Beckman Coulter, Brea, CA). Synergism of combinational drug treatments was determined by calculating the combination index (CI) using CalcuSyn Version 2.0 (Biosoft, Ferguson, MO) as described [46]. Statistical significance was determined by unpaired Student’s t test using GraphPad PRISM version 5.0c (San Diego, CA).

### Construction of stable NALM6 cell lines expressing shRNAs

Stable NALM6 cells expressing shRNAs against CHOP, ERO1, STIM1, Orai1, and BIM were generated using lentiviral particles obtained from Santa Cruz Biotechnology (Dallas, TX) and transduced by spinoculation as described [8]. Stably transduced ALL cells were selected in presence of 1 µg/mL puromycin, and shRNA-mediated down-regulation validated by western blots. Plasmids encoding Orai1-YFP (Addgene #19756), and STIM1-CFP (Addgene #19755) were obtained from Addgene, and transfected in NALM6 cells by nucleofection and selected in presence of G418 and puromycin, respectively, as described [47]. NALM6 cells expressing YFP and CFP proteins were sorted based on YFP and CFP fluorescent signals using BD FACS SORP Aria-II high-speed cell sorter.

### Immunoblots and co-immunoprecipitation (Co-IP) assays

Proteins were extracted using 1X RIPA buffer supplemented with protease/phosphatase inhibitor cocktail (Thermo Fisher Scientific Inc., Waltham, MA), and concentration determined using the BCA protein assay kit (Thermo Fisher Scientific Inc., Waltham, MA). Immunoblots (western blot) were performed as previously described [48]. Primary antibodies against ERK1/2, p-ERK1/2 (Thr202/Tyr204), MEK1/2, p-MEK1/2 (Ser217/221), p-PKCα/β II (Thr638/641), CAMKII, p-CAMKII (Thr286), BIM; p-BIM (Ser69), CHOP, Bcl-2, Bcl-xL, p-eIF2α (Ser51), STIM1, PARP, NEDD8 and secondary HRP-conjugated antibodies were obtained from Cell Signaling (Danvers, MA). Antibodies for PKCβ, luciferase, ERO1 and Orai1 were purchased from Santa Cruz Biotechnology (Dallas, TX). Proteins expression was determined by densitometry analysis of the immune-detected bands, normalized to β-actin, and presented relative to control (fold induction). Co-IP assays were carried out using the Pierce™ Classic Magnetic IP Kit (Thermo Fisher Scientific Inc., Waltham, MA) as described [49].

### Determination of intracellular Ca^2+^

Intracellular Ca^2+^ was measured using the Fluo-8 No Wash Ca^2+^ Assay Kit (Abcam, Cambridge, MA), a method previously validated by others [50-52]. Briefly, NALM6 cells were incubation with pevonedistat (400 nM) or tunicamycin (400 ng/mL) for 24 h, washed twice with HHBS (1X Hank’s with 20 mM Hepes Buffer, pH 7.0), and seeded (200,000 cells/well in a volume of 100 µL) in 96-well plate with black wall and clear flat bottom. Then, 100 µL of Fluo-8 dye solution was added to each well and plates were incubated for 30 min at 37°C, followed by an additional incubation of 30 min at room temperature. The fluorescence intensity was measured at Ex/Em = 490/525 nm. Thapsigargin (TG, 10 µM) was added to each well and kinetic measured at 27 sec intervals for 20 min (n = 3). For Ca^2+^ measurement using microscopic immunofluorescence, cells were treated with BTP-2 (10 µM) or pevonedistat (400 nM) either alone or in combination (pevonedistat + BTP-2) for 24 h, washed twice with HHBS, and centrifuged at 1000 rpm for 5 min. Fluorescent cell images were captured using a Leica immunofluorescence microscope with a 10× magnification.

### Confocal imaging analysis

NALM6-Orai1-YFP expressing cells were treated with vehicle (DMSO) or pevonedistat (400 nM) for 24 h, washed with PBS, and transferred onto coverslips. Then, plates were sealed using nail polish. Images were captured using a Leica SP5 spectral confocal inverted microscope with a 60× magnification, and analyzed using Leica LAS X software.

### Mouse ALL xenograft and bioluminescent imaging

ALL cells engraftment in NSG mice, bioluminescent imaging and leukemia burdens were carried out as previously described [4]. Once leukemia xenograft confirmed by either flow cytometry (∼1% of human ALL cells CD10/CD19) or bioluminescent signal (≥1.05x103 photons/second/cm2/steradian, p/s/cm2/sr), mice were randomly assigned to four treatment groups (6 mice per group): pevonedistat (s.c., 66 mg/kg in 20% hydroxypropyl-β-cyclodextrin (HPbCD, Onbio Inc., Ontario, Canada)); selumetinib (SEL, p.o., 50 mg/kg in PBS with less than 3% DMSO); combination treatment of both compounds (pevonedistat plus SEL), and the Control group (vehicles). The agents were administered twice daily during weekdays and once per day during weekends for up to 60 days. For biological references, spleens from deceased animals were extracted, lymphocytes isolated, and protein extracts processed for Western blot analysis as described [4]. All experiments were conducted under the supervision of the University of Miami Institutional Animal Care and Use Committee.

## References

[1] Pui CH, Robison LL, Look AT (2008). Acute lymphoblastic leukaemia. Lancet.

[2] Rowe JM, Goldstone AH (2007). How I treat acute lymphocytic leukemia in adults. Blood.

[3] Pui CH, Carroll WL, Meshinchi S, Arceci RJ (2011). Biology, risk stratification, and therapy of pediatric acute leukemias: an update. J Clin Oncol.

[4] Leclerc GM, Zheng S, Leclerc GJ, DeSalvo J, Swords RT, Barredo JC (2016). The NEDD8-activating enzyme inhibitor pevonedistat activates the eIF2alpha and mTOR pathways inducing UPR-mediated cell death in acute lymphoblastic leukemia. Leuk Res.

[5] Petroski MD, Deshaies RJ (2005). Function and regulation of cullin-RING ubiquitin ligases. Nat Rev Mol Cell Biol.

[6] Soucy TA, Smith PG, Milhollen MA, Berger AJ, Gavin JM, Adhikari S, Brownell JE, Burke KE, Cardin DP, Critchley S, Cullis CA, Doucette A, Garnsey JJ (2009). An inhibitor of NEDD8-activating enzyme as a new approach to treat cancer. Nature.

[7] Kharabi Masouleh B, Geng H, Hurtz C, Chan LN, Logan AC, Chang MS, Huang C, Swaminathan S, Sun H, Paietta E, Melnick AM, Koeffler P, Muschen M (2014). Mechanistic rationale for targeting the unfolded protein response in pre-B acute lymphoblastic leukemia. Proc Natl Acad Sci U S A.

[8] DeSalvo J, Kuznetsov JN, Du J, Leclerc GM, Leclerc GJ, Lampidis TJ, Barredo JC (2012). Inhibition of Akt potentiates 2-DG-induced apoptosis via downregulation of UPR in acute lymphoblastic leukemia. Mol Cancer Res.

[9] Irving J, Matheson E, Minto L, Blair H, Case M, Halsey C, Swidenbank I, Ponthan F, Kirschner-Schwabe R, Groeneveld-Krentz S, Hof J, Allan J, Harrison C (2014). Ras pathway mutations are prevalent in relapsed childhood acute lymphoblastic leukemia and confer sensitivity to MEK inhibition. Blood.

[10] Perentesis JP, Bhatia S, Boyle E, Shao Y, Shu XO, Steinbuch M, Sather HN, Gaynon P, Kiffmeyer W, Envall-Fox J, Robison LL (2004). RAS oncogene mutations and outcome of therapy for childhood acute lymphoblastic leukemia. Leukemia.

[11] Knight T, Irving JA (2014). Ras/Raf/MEK/ERK Pathway Activation in Childhood Acute Lymphoblastic Leukemia and Its Therapeutic Targeting. Front Oncol.

[12] Roberts PJ, Der CJ (2007). Targeting the Raf-MEK-ERK mitogen-activated protein kinase cascade for the treatment of cancer. Oncogene.

[13] Chuderland D, Seger R (2008). Calcium regulates ERK signaling by modulating its protein-protein interactions. Commun Integr Biol.

[14] Feske S (2007). Calcium signalling in lymphocyte activation and disease. Nat Rev Immunol.

[15] Stathopulos PB, Schindl R, Fahrner M, Zheng L, Gasmi-Seabrook GM, Muik M, Romanin C, Ikura M (2013). STIM1/Orai1 coiled-coil interplay in the regulation of store-operated calcium entry. Nat Commun.

[16] Park CY, Hoover PJ, Mullins FM, Bachhawat P, Covington ED, Raunser S, Walz T, Garcia KC, Dolmetsch RE, Lewis RS (2009). STIM1 clusters and activates CRAC channels via direct binding of a cytosolic domain to Orai1. Cell.

[17] Krueger F, Madeja Z, Hemberger M, McMahon M, Cook SJ, Gaunt SJ (2009). Down-regulation of Cdx2 in colorectal carcinoma cells by the Raf-MEK-ERK 1/2 pathway. Cell Signal.

[18] Wen-Sheng W (2006). Protein kinase C alpha trigger Ras and Raf-independent MEK/ERK activation for TPA-induced growth inhibition of human hepatoma cell HepG2. Cancer Lett.

[19] Ueda Y, Hirai S, Osada S, Suzuki A, Mizuno K, Ohno S (1996). Protein kinase C activates the MEK-ERK pathway in a manner independent of Ras and dependent on Raf. J Biol Chem.

[20] Chen YB, LaCasce AS (2008). Enzastaurin. Expert Opin Investig Drugs.

[21] Mochly-Rosen D, Das K, Grimes KV (2012). Protein kinase C, an elusive therapeutic target?. Nat Rev Drug Discov.

[22] Anderson ME (2005). Calmodulin kinase signaling in heart: an intriguing candidate target for therapy of myocardial dysfunction and arrhythmias. Pharmacol Ther.

[23] Li G, Mongillo M, Chin KT, Harding H, Ron D, Marks AR, Tabas I (2009). Role of ERO1-alpha-mediated stimulation of inositol 1,4,5-triphosphate receptor activity in endoplasmic reticulum stress-induced apoptosis. J Cell Biol.

[24] Buckley BJ, Whorton AR (1997). Tunicamycin increases intracellular calcium levels in bovine aortic endothelial cells. Am J Physiol.

[25] Lytton J, Westlin M, Hanley MR (1991). Thapsigargin inhibits the sarcoplasmic or endoplasmic reticulum Ca-ATPase family of calcium pumps. J Biol Chem.

[26] Thastrup O, Cullen PJ, Drobak BK, Hanley MR, Dawson AP (1990). Thapsigargin, a tumor promoter, discharges intracellular Ca^2+^ stores by specific inhibition of the endoplasmic reticulum Ca2(+)-ATPase. Proc Na tl Acad Sci USA.

[27] Korfi K, Smith M, Swan J, Somervaille TC, Dhomen N, Marais R (2016). BIM mediates synergistic killing of B-cell acute lymphoblastic leukemia cells by BCL-2 and MEK inhibitors. Cell Death Dis.

[28] Ma G, Wei M, He L, Liu C, Wu B, Zhang SL, Jing J, Liang X, Senes A, Tan P, Li S, Sun A, Bi Y (2015). Inside-out Ca(2+) signalling prompted by STIM1 conformational switch. Nat Commun.

[29] Hodeify R, Selvaraj S, Wen J, Arredouani A, Hubrack S, Dib M, Al-Thani SN, McGraw T, Machaca K (2015). A STIM1-dependent ‘trafficking trap’ mechanism regulates Orai1 plasma membrane residence and Ca(2)(+) influx levels. J Cell Sci.

[30] Hoover PJ, Lewis RS (2011). Stoichiometric requirements for trapping and gating of Ca^2+^ release-activated Ca^2+^ (CRAC) channels by stromal interaction molecule 1 (STIM1). Proc Natl Acad Sci U S A.

[31] Vattem KM, Wek RC (2004). Reinitiation involving upstream ORFs regulates ATF4 mRNA translation in mammalian cells. Proc Natl Acad Sci U S A.

[32] Palam LR, Baird TD, Wek RC (2011). Phosphorylation of eIF2 facilitates ribosomal bypass of an inhibitory upstream ORF to enhance CHOP translation. J Biol Chem.

[33] Boyce M, Bryant KF, Jousse C, Long K, Harding HP, Scheuner D, Kaufman RJ, Ma D, Coen DM, Ron D, Yuan J (2005). A selective inhibitor of eIF2alpha dephosphorylation protects cells from ER stress. Science.

[34] Barbosa C, Peixeiro I, Romao L (2013). Gene expression regulation by upstream open reading frames and human disease. PLoS Genet.

[35] Steelman LS, Franklin RA, Abrams SL, Chappell W, Kempf CR, Basecke J, Stivala F, Donia M, Fagone P, Nicoletti F, Libra M, Ruvolo P, Ruvolo V (2011). Roles of the Ras/Raf/MEK/ERK pathway in leukemia therapy. Leukemia.

[36] Kim I, Xu W, Reed JC (2008). Cell death and endoplasmic reticulum stress: disease relevance and therapeutic opportunities. Nat Rev Drug Discov.

[37] Starck SR, Tsai JC, Chen K, Shodiya M, Wang L, Yahiro K, Martins-Green M, Shastri N, Walter P (2016). Translation from the 5’ untranslated region shapes the integrated stress response. Science.

[38] Araki K, Nagata K (2011). Protein folding and quality control in the ER. Cold Spring Harb Perspect Biol.

[39] Zhou X, Tan M, Nyati MK, Zhao Y, Wang G, Sun Y (2016). Blockage of neddylation modification stimulates tumor sphere formation *in vitro* and stem cell differentiation and wound healing *in vivo*. Proc Natl Acad Sci U S A.

[40] Ley R, Balmanno K, Hadfield K, Weston C, Cook SJ (2003). Activation of the ERK1/2 signaling pathway promotes phosphorylation and proteasome-dependent degradation of the BH3-only protein, Bim. J Biol Chem.

[41] Li H, Tan M, Jia L, Wei D, Zhao Y, Chen G, Xu J, Zhao L, Thomas D, Beer DG, Sun Y (2014). Inactivation of SAG/RBX2 E3 ubiquitin ligase suppresses KrasG12D-driven lung tumorigenesis. J Clin Invest.

[42] Leclerc GJ, York TA, Hsieh-Kinser T, Barredo JC (2007). Molecular basis for decreased folylpoly-gamma-glutamate synthetase expression in a methotrexate resistant CCRF-CEM mutant cell line. Leuk Res.

[43] Godbersen JC, Humphries LA, Danilova OV, Kebbekus PE, Brown JR, Eastman A, Danilov AV (2014). The Nedd8-activating enzyme inhibitor MLN4924 thwarts microenvironment-driven NF-kappaB activation and induces apoptosis in chronic lymphocytic leukemia B cells. Clin Cancer Res.

[44] Messinger YH, Gaynon PS, Sposto R, van der Giessen J, Eckroth E, Malvar J, Bostrom BC, Therapeutic Advances in Childhood Leukemia & Lymphoma (TACL) Consortium (2012). Bortezomib with chemotherapy is highly active in advanced B-precursor acute lymphoblastic leukemia: Therapeutic Advances in Childhood Leukemia & Lymphoma (TACL) Study. Blood.

[45] Swords RT, Erba HP, DeAngelo DJ, Bixby DL, Altman JK, Maris M, Hua Z, Blakemore SJ, Faessel H, Sedarati F, Dezube BJ, Giles FJ, Medeiros BC (2015). Pevonedistat (MLN4924), a First-in-Class NEDD8-activating enzyme inhibitor, in patients with acute myeloid leukaemia and myelodysplastic syndromes: a phase 1 study. Br J Haematol.

[46] Chou TC (2006). Theoretical basis, experimental design, and computerized simulation of synergism and antagonism in drug combination studies. Pharmacol Rev.

[47] Leclerc GJ, DeSalvo J, Du J, Gao N, Leclerc GM, Lehrman MA, Lampidis TJ, Barredo JC (2015 Aug 20). Mcl-1 downregulation leads to the heightened sensitivity exhibited by BCR-ABL positive ALL to induction of energy and ER-stress. Leuk Res.

[48] Kuznetsov JN, Leclerc GJ, Leclerc GM, Barredo JC (2011). AMPK and Akt determine apoptotic cell death following perturbations of one-carbon metabolism by regulating ER stress in acute lymphoblastic leukemia. Mol Cancer Ther.

[49] Leclerc GJ, Sanderson C, Hunger S, Devidas M, Barredo JC (2010). Folylpolyglutamate synthetase gene transcription is regulated by a multiprotein complex that binds the TEL-AML1 fusion in acute lymphoblastic leukemia. Leuk Res.

[50] Eitan E, Hutchison ER, Marosi K, Comotto J, Mustapic M, Nigam SM, Suire C, Maharana C, Jicha GA, Liu D, Machairaki V, Witwer KW, Kapogiannis D (2016). Extracellular Vesicle-Associated Abeta Mediates Trans-Neuronal Bioenergetic and Ca(2+)-Handling Deficits in Alzheimer’s Disease Models. NPJ Aging Mech Dis.

[51] Ishii M, Rohrer B (2017). Bystander effects elicited by single-cell photo-oxidative blue-light stimulation in retinal pigment epithelium cell networks. Cell Death Discov.

[52] Albrecht T, Zhao Y, Nguyen TH, Campbell RE, Johnson JD (2015). Fluorescent biosensors illuminate calcium levels within defined beta-cell endosome subpopulations. Cell Calcium.

